# Overview of current adipose-derived stem cell (ADSCs) processing involved in therapeutic advancements: flow chart and regulation updates before and after COVID-19

**DOI:** 10.1186/s13287-020-02006-w

**Published:** 2021-01-04

**Authors:** Loubna Mazini, Mohamed Ezzoubi, Gabriel Malka

**Affiliations:** 1 Laboratoire Cellules Souches et Régénération Cellulaire et Tissulaire, Center of Biological and Medical Sciences CIAM, Mohammed VI Polytechnic University (UM6P), Lot 660, Hay Moulay Rachid, 43150 Ben Guerir, Morocco; 2 Centre des Brûlés et chirurgie réparatrice, Centre Hospitalier Universitaire Ibn Rochd Casablanca, Faculté de Médecine et de Pharmacie Casablanca, Casablanca, Morocco

**Keywords:** ADSCs, HCT/P 351-361, ATMPs, cGMP, Standardization, COVID-19

## Abstract

Adipose-derived stem cells (ADSCs) have raised big interest in therapeutic applications in regenerative medicine and appear to fulfill the criteria for a successful cell therapy. Their low immunogenicity and their ability to self-renew, to differentiate into different tissue-specific progenitors, to migrate into damaged sites, and to act through autocrine and paracrine pathways have been altogether testified as the main mechanisms whereby cell repair and regeneration occur. The absence of standardization protocols in cell management within laboratories or facilities added to the new technologies improved at patient’s bedside and the discrepancies in cell outcomes and engraftment increase the limitations on their widespread use by balancing their real benefit versus the patient safety and security. Also, comparisons across pooled patients are particularly difficult in the fact that multiple medical devices are used and there is absence of harmonized assessment assays despite meeting regulations agencies and efficient GMP protocols. Moreover, the emergence of the COVID-19 breakdown added to the complexity of implementing standardization. Cell- and tissue-based therapies are completely dependent on the biological manifestations and parameters associated to and induced by this virus where the scope is still unknown. The initial flow chart identified for stem cell therapies should be reformulated and updated to overcome patient infection and avoid significant variability, thus enabling more patient safety and therapeutic efficiency. The aim of this work is to highlight the major guidelines and differences in ADSC processing meeting the current good manufacturing practices (cGMP) and the cellular therapy-related policies. Specific insights on standardization of ADSCs proceeding at different check points are also presented as a setup for the cord blood and bone marrow.

## Introduction

Adipose tissue (AT) was first used as a grafting tool in plastic surgery. Freshly isolated from AT, stromal vascular fraction (SVF) was used for more suitable satisfactory tissue regeneration as it contains multipotent stem/stromal cells widely reported for their proliferative and differentiation behavior called adipose-derived stem cells (ADSCs). These cells are mesenchymal stem cells (MSCs) and were used both for their ability to differentiate into cells belonging to mesodermic, endodermic, and exodermic cell-specific lineages and for their paracrine activity [1–3]. When transplanted into damaged sites, these cells are able to interact with their adjacent microenvironment leading to the generation of new committed progenitors and cells. At the same way, they secrete exosomes containing growth factors, cytokines, chemokines, and micro-RNA involved in restoring tissue defects and biological functions [4–11]. These biomolecules play a crucial role through stimulation of the molecular mechanisms involved in angiogenesis, immunomodulation, and cell proliferation/differentiation whereby repair of damaged tissue occurs. ADSCs were reported as better immunomodulatory actors lacking MHC class II opening the way to therapeutic investigations at allogenic setting. Increasing evidences also argue that their immunomodulatory effect is related to their regenerative ability [12]. Interestingly, MSCs produced molecules with antimicrobial activity reducing pain, making them a promising tool against infections and cytokine storm [13–16]. Moreover, isolated from different origins, MSC-derived exosomes are reported efficient and promising immunomodulators in treating ill COVID-19 patients [17–28]. Altogether, these characteristics have emphasized ADSC use as an effective approach in the treatment of patients suffering from COVID-19 [14, 29, 30].

ADSCs are mainly separated from SVF using a mechanical or enzymatic process, seeded facultatively in an expansion culture before being administered through autologous or allogenic transplantation. Their use in therapeutic protocols is conditioned by high cell numbering, their low culturing passage, and reduced time delay before processing. On the other hand, their therapeutic benefit is mandatory by the proliferative potency and ability to differentiate into cell tissue of interest after administration. Nevertheless, their clinical outcomes might be hampered by viral molecules released within their exosomes complicating patient safety and security associated to the success of this cell-free therapy in regenerative medicine [31].

## ADSC therapeutic use: patient safety and regulatory framework

ADSC therapy has proven efficacy and efficiency and holds great promise in regenerative medicine. Positive benefit-risk in restoring wound defects, bone regeneration, and autoimmune and neurodegenerative diseases has been reported [32–41]. However, some serious side effects have been shown such as blindness in SVF-treated patients presenting macular degeneration [42], challenging the justification of this cell therapy. Thus, patient safety and security have become the critical parameter controlling the widespread use and the bringing of stem cell-based products to the market. Actually, there are no universal guidelines for assessing a biological product, especially those classified as “non-homologous use” [43].

To ensure patient safety and security, regulation agencies continuously modify and reinforce their approaches to regulate cellular and tissue-based products. The “human cells, tissues, or cellular or tissue-based products” (HCT/P) regulation has been set up by the Food and Drug Administration (FDA) based on two major criteria: (i) their minimal manipulation and (ii) the homologous use for the tissue-based products. Accordingly, an HCT/P product meets the criteria of the regulatory requirements 21 CFR 1271 section 361 called HCT/P-361. These products do not need FDA approval for release from the facility and post-marketing thanks to the absence of other articles excepting water, crystalloids, sterilizing, or preserving agents. In addition, the HCT/P-361 regulation framework anticipates that the HCT/P should have or not have a systemic effect and a dependence upon the metabolic activity of the living cells for its primary function. They must also comply with an autologous, first- or second-degree allogenic use. Other cell and tissue products are cannot be considered lawful by the HCT/P-361 requiring thus the 21 CFR 1271 section 351 regulation and the FDA approval as a Biologics License Application (BLA). Accordingly, SVF appears to fulfill all the criteria of the HCT/P-361; however, the use of collagenase to digest AT and its presence in the separated fraction has led to its recent classification under HCT/P-351. ADSCs have been similarly classified HCT/P-351.

To limit the processing-associated risks, several programs such as the American Association of Blood Banks (AABB), NetCord, the Foundation for the Accreditation of Cellular Therapy (FACT), and the Joint Accreditation Committee (JACIE) have drawn and designed specific accreditations to better manage the stem cell facilities and banks. In this technical aspect, these recommendations established a uniform level of practice aiming to promote high-quality products. The good tissue practice (GTP) rule proposed also under HCT/Ps rules forms the principal elements of their harmonization framework. These GTPs are intended to prevent HCT/Ps from contamination with infectious pathogens and to ensure their integrity and function through maintaining high quality and safety standards. The good manufacturing practice (GMP) is also designated to track and follow the level of processing and manufacturing of each cell product. However, the GMP “grade” is always thought more important for obtaining regulatory approval [44].

In Europe, the European Medicines Agency (EMA) is the authority in charge of evaluating and approving all regenerative medicine products. Their directive statutes on the advanced therapy medicinal products (ATMPs) are ensuring preclinical testing under good laboratory practice (GLP) similarly to the USA. The ATMP should meet the quality standards for intended use, traceability requirement, risk management system, and especially clinical approval to be marketed under GMP conditions.

Despite satisfying these requirements, discrepancies in the therapeutic outcomes are reported, due to the variability in medical devices and reagents used for processing and quality assessment.

## State of the art of the GMP requirements for ADSC use

Widespread autologous and allogeneic ADSC use is undeniably reliable to large-scale manipulation with appropriate assurance and quality control in compliance with cGMP. High numbers of viable and functional fresh or cryopreserved ADSCs intended for clinical use are usually required. Technical and medical issues relative to the collection of tissue of origin, isolation, and storage of these cells have become thus paramount in devising the GMP conditions for future clinical use. According to the current cellular therapy regulations, ADSC preparations and derivatives should meet the GMP requirements including raw materials, clinical-grade reagents, and stem cell facilities to achieve and assure quality and safety during the entire process of preparation, banking, and manufacturing [45, 46].

On one side, basic cell processing methods appeared unqualified to be transferred into a cGMP facility; the whole process is considered from tissue and cell collection, manipulation, storage, and releasing to the point of care. On the other side, clinical-grade or cGMP-compatible reagents should be used for more safety in processing and testing. If not available, these reagents should be justified to prevent the potential risk of transferring immunogenic xenoproteins, infectious agents, or any other animal species leading thus the legibility of the cell product. A specific concern to highly consider is the animal-containing products deriving from animals being potentially infected or hosted by the coronavirus. Additional and rigorous actions should be performed to completely secure these products’ innocuity.

Some reagents are now available on the market free from animal proteins and developed at GMP grade such as Ficoll-Paque TM PREMIUM (GE Healthcare Life Science, USA), Collagenase-NB6 (Serva Electrophoresis GmbH, Germany), Celase™, Adipase® or GIDzyme-2 (GID Inc), TrypLE TM Select (Inviotrogen) and TrypZean (Sigma-Aldrich), phosphate-buffered saline (PBS) and dimethyl sulfoxide (DMSO) (CliniMACS, Miltenyi Biotech, Germany), human platelet lysates PLT-Max (Mill Creek), Stemulate™ PL-S, and Stemulate™ PL-SP (COOK General Biotechnology).

Despite the application of HCT/P guidelines and regulations, manipulations of ADSCs and their derivatives showed many differences in their reproducibility and efficiency between laboratories and facilities and still need to be thoroughly well documented and listed according to specific standardized frameworks.

## SVF processing

**Fig. 1:**
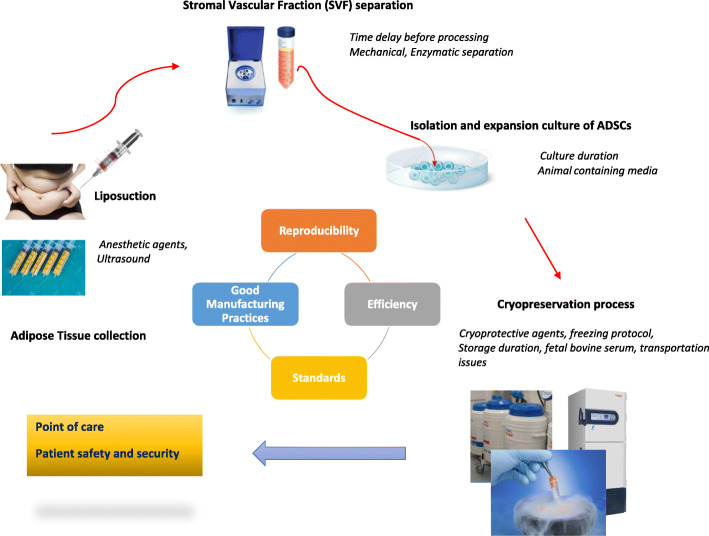
Processing of adipose tissue. Lipoaspirates are collected using draining agents and undergo mechanical and/or enzymatical procedure to separate the stromal vascular fraction (SVF). ADSCs are then isolated mostly by seeding SVF in culture and expanded before releasing to the point of care. Cryopreservation ensures storage in liquid nitrogen before using in clinical issues. This manual flow chart has been improved by using all-in-one single-use kits and commercialized medical devises in completely closed or semi-closed systems to meet the good manufacturing practices (GMP) requirements for therapeutic use of stem cells and derivatives

Defined methods were shown to harvest AT with regard to lipoaspirate’s invasive manner, cell viability, and collection volume [47–49]. Figure 1 summarizes the basically steps involved in SVF and ADSC processing. After Coleman’s protocol, semi-closed and completely closed systems aiming to separate fat tissue fraction from the contaminating fluids (saline solution, adrenaline, anesthetics) have been elaborated to completely secure the compelling process mandatory for clinical use. Studies have indicated that local anesthetic agents might negatively impact quantitatively and qualitatively preadipocytes [49]. Indeed, reduced ADSC viability and chondrocyte cytotoxicity were shown after lidocaine [50–52]. Ultrasound-assisted liposuction has also been reported to compromise recovery and expansion of ADSCs [53], suggesting that the absence of a standardized fat harvesting process is the first parameter leading to variability in ADSC therapeutic outcomes.

To improve SVF separation and comply with the GMP requirements, medical devices and highly technological equipment have been developed. These commercially available devices consisted usually of specific bags for washing AT and permitting the enzymatic digestion and SVF separation using at the same time clinical-grade reagent exempt from animal additives. Cell outcomes and viability added to their clonogenic potencies are reported significantly efficient compared to the control separation without preventing the variability related to the manipulator [54–57].

These devices provide all the supplies needed for the SVF separation in all-in-one single-use kit having the advantage to reduce the risk of contamination during cell separation and favoring their use at patient’s bedside [58, 59]. Identical system has been used for the bone marrow (BM) with adapted perfusion system leading to expansion of BM regenerative cells [60] and MSCs [61–63].

Table 1 represents the main commercialized systems used to separate SVF in semi-automated or fully automated level. Compared to the manual methods requiring standard laboratory equipment, most of these systems allow an improvement in SVF yield process and limit the use of biosafety hood, centrifuge, and heather shaker by increasing their automation and simplifying the multiple step of the process. Semi-automated devices might need user intervention. This is completely carried out when performing the entire process in a single closed system only requiring the insertion of the lipoaspirates. However, increasing automation leads to increasing of disposable costs. Additionally, some devices are purchased with their GMP-grade proteolytic enzymes associated to their single-use kit such as the GID-SVF system (Table 1) [56, 57, 64–67]. In the same way, devices offering mechanical methods are less expensive but time consuming than those using enzymatic methods and requiring specific GMP-grade reagents and enzymes.

**Table 1:** Point of care devices for bed side separation of SVF

Medical devise	Manufacturer	Closed/semi-closed	Separation method	Proceeded fat quantity (g)	Process duration (min)	Cell yield/g of fat Device/control (D/C)	Cell viability D/C or D	References
Celution R 800/CRS StemSource 900/MB	Cytori Therapeutics, Inc	Closed	Enzymatic	300 g	90	2.41 × 10^6^/NP	NP	[57, 58]
GID SVF-1, SVF-2	GID Group, Inc	Closed	Enzymatic	300 g	90	0.425 × 10^6^ ± 0.047 × 10^6^/0.795 × 10^6^ ± 0.228 × 10^6^	50–84%	[56, 57, 64–67]
Icellator®	Tissue Genesis, Inc.	Closed	Enzymatic	60	80	0.25–2.0 × 10^6^	64.5 ± 11.4	[57, 68, 69]
Puregraft™	Eurosilicone	Semi-closed	Mechanical	250	100	0.25 × 10^6^ ± 0,07 × 10^6^/0.79 × 10^6^ ± 0.228 × 10^6^	77%	[57]
Stem.pra®	Proteal	Closed	Mechanical	200	110	0.535 × 10^6^ ± 0.209 × 10^6^/0.795 × 10^6^ ± 0.228 × 10^6^	69%	[57]
Cha-Station™	PNC International Co., Ltd.	Semi-closed	Mechanical Enzymatic	200	90	0.05 × 10^6^	NA	[57, 70]
Sepax®	Biosafe Grop SA	Semi-closed	Mechanical	300	90–120	2.6 ± 1.2 × 10^5^/ML	NA	[55, 57]
UNISTATION™	NeoGenesis	Closed	Not provided	NP	NP	1.1 × 10^5^ ± 1.1 × 10^5^/2.0 × 10^5^ ± 1.7 × 10^5^	NA	[71]
Lipokit MaxStem	Medi-Khan’s	Semi-closed	Enzymatic	NP	88–120	0.35 × 10^6^	50–84	[56, 70]

Processing with the different devices is reported regarding the closed or semi-closed systems and their efficiency to separate SVF in terms of fat quantity, cell yield and viability, and duration. These devices provide all-in-one single-use kit for the SVF separation and might also contain their GMP-grade proteolytic enzymes. *NP* not provided and, *NA* not applicable

Table 1 also shows the higher SVF yield per gram fat for the Celurion and Icellator devices both performing enzymatic separation in a closed system with preferentially 5x hold more processed fat by the Celurion system associated to a higher disposable cost [57]. In addition, the Celurion 800/CRS device presents less residual enzyme levels than that observed with the Cha-Station and Lipokit systems [70] while a similar negligible residual collagenase was reported by Aronowitz et al. for the Lipokit, GID-SVF-2, and StemSource 900/MB Celution system [56, 70].

Nevertheless, some findings reported automated system limitations regarding efficacy and cell outcomes [68]. However, the colony-forming unit fibroblast (CFU-F) assay used as the overall indicator of the ADSC frequency and proliferation ability indicate an improved colony-forming efficiency with the GID-SVF1 method when compared to Puregraft and Stem.pras with no significance regarding the reference method [57]. CFU-F frequency was also differently reported for the Lipokit, GID-SVF-2, and the Cytori systems [56] demonstrating the existence of consistent variability within the identification of the advantageous system in terms of cellular benefit and device practicability. The absence of standardizing separation protocols performed in the different laboratories added to the complexity of the widespread use of these commercialized systems in an approved clinical use. Some systems including Station Beauty Cell, Stempia kit (N-Biotek Inc.), and Kanaka working station for SVF separation and washing (Kanaka.co.jp) are commercialized without any scientific or preclinical support and used as automated processing at patient’s bedside.

Although these systems generated a significant variability of SVF regarding ADSC’s profile, the GID-SVF-1, Stem.pras, and Puregraft did not impact CD73 and HLA-ABC expression level similarly to the hematopoietic markers CD14, CD45, HLADR, and CD34 when compared to the reference method. Nevertheless, CD34 expression was drastically decreased in passage 1 expanded ADSCs [57]. Aronowitz et al. reported no significant differences in the frequency of CD31−/CD34+/CD45 cells in SVF separated by Lipokit, GID SVF-2, and StemSource 900/MB systems [56]. When using the Unistation device, SVF presented a decreased CD34+ expression frequency and an increased CD45+ cell counts with highly proliferative CD271 similar to the reference method [71]. On the other side, the Celution system reported significantly more endothelial cells and CD34/CD31 cells [70]. A recent report on the use of Icellator system demonstrated a predominant expression of CD90, CD29, and CD34 on cryopreserved SVF followed by CD45, CD105, CD73, and CD44; however, comparative analysis with a reference method was not reported [69]. Inversely, isolated by the Stem.pras method, SVF was decreased significantly in their CD90 expression level [57]. This suggests that in the absence of a comparative analysis and a standardized assessment method of the different devices, large clinical use of AT-derived products associated with patient’s benefit and safety meeting the international requirements remains limited. Side-by-side clinical trials will be required to establish the relevance of these differences.

## ADSC separation and expansion

Different protocols were proposed in isolating ADSCs [45, 72–79]. Increasing adherent ADSCs by taking SVF to 60 h adhesion followed by forceful washing was also used [80]. Although sorting among specific cell markers resulted in subpopulations exhibiting distinct osteogenic and chondrogenic differentiation potentials [81, 82]. Other reports have proposed CD271 as a marker for tissue regeneration and showed that CD271+ ADSCs presented differentiation ability to mesodermic lineages [83, 84].

ADSC separation is preferentially performed by prolonged culture of the SVF mixture over time by their adherence to plastic and successive culture passages [45, 74, 77–79]. Lately, culture passages contained ADSCs populations with a stromal phenotype associated with homing properties. In contrast, early passages represented heterogeneous ADSC populations with distinct surface-cell adhesion molecules consistent with their stemness and differentiation abilities [79, 85, 86].

Despite complying with GMP conditions, culture medium composition is a key regulator in defining cell immunophenotype and secretome composition in proteins and nucleic acids [4, 11, 54, 87]. There are evidences that ADSCs were primed with their microenvironment’s composition highlighting the need of standardized ready-to-use culture media. Basically, culture medium is composed of Dulbecco’s minimal essential medium (DMEM) supplemented with 10% of fetal bovine serum (FBS), fetal calf serum (FCS), human platelet lysate (hPL), or growth factors derived from platelets. One of them, the fibroblast growth factor-2 (FGF-2), specifically improves chondrogenic differentiation [88] and together with FBS induced highly expression of the CD146 antigens by cultured ADSCs [87]. Using animal products containing reagents remains a real concern through their contaminating biomolecules.

These distinct properties are promising and might have important implications for the pre-selection of ADSC subpopulations and thus sibling cell-based therapies. Thus, such expanded subpopulations might be reproducibly manufactured in a time point for a clinical specific use.

Many standardized methods of isolating ADSCs have been proposed and designed, consisting of mechanical (centrifuge) or enzymatic (collagenase) means or both of them in a complete closed system [57, 75, 89–92]. These approaches aim to prevent the conventional methods associated to contamination risks and time consuming and specifically to reduce manufacturing time and costs. Indeed, these closed or semi-closed devices are effective, safe, and economic for a large clinical use and allowed for greater number of isolated and expanded ADSCs when compared to the manual procedure [76, 89, 90, 92]. The Quantum Cell Expansion System is a hollow fiber bioreactor (Terumo BCT, Lakewood, CO, USA) mostly used due to the reproducible, economical, and the large cell quantities seeded. This system is a GMP-compliant able to generate 1 × 10^8^ cells in 2 weeks [93]. Similar efficient, however, manual protocols have been reported in compliance with GMP conditions [45, 76, 89].

Thoroughly, expansion protocols of ADSCs have been improved by using PL with more benefit than FBS regarding proliferation and differentiation capacities, CFU-F frequency, and cell senescence [87, 94–101]. Using the Quantum Cell Expansion System, ADSCs yield estimated as population doubling is significantly more important than in control conditions with FBS [90, 92]. Also, when expanded with PL, ADSCs exhibited less immunogenic potential with a preserved normal karyotype for at least six passages of culture [102]. Likewise, evidences have been demonstrated for platelet-rich plasma (PRP) in inducing proliferation and motility of ADSCs without affecting survival of mature adipocytes improving thus fat engraftment outcomes [103]. Added to that, expansion protocols are set up for different passages and the literature showed inconsistent results. We have already reported that ADSCs should not be expanded more than 2 passages before being used or frozen [4] due to the modified immunological profile. All these factors raised the need to formulate a standard operating protocol complying with GMP requirements for extensive clinical scale.

## Cryopreservation and storage

### Freezing media

Basically, cryopreservation could not occur without using cryoprotective agents (CPA) alone or in combination with FBS thanks to their improvement of cell viability of frozen and thawed cells. Several hydrocolloids and organic osmolytes are successfully used as CPA [52, 104–108]. Because of storage of SVF or ADSCs is usually performed with FBS, many studies aimed to develop efficient cryopreservation methods without serum and animal products to avoid immunological reactions and risk of transmission of bacterial/viral infections and prions. Xeno- and serum-free media were thus formulated objectively to be used for isolation, expansion, and banking of ADSCs. In these media, CPAs were supplemented with polymers and anti-oxidants to mimic the beneficial effects of serum [105, 109–111]. Consequently, the recovery, functionality, and multipotency of thawed ADSCs appeared fully maintained [105, 110, 111].

However, there is evidence that the cellular activity of ADSCs after freezing and thawing was affected by CPA [112]. The most used one remains the DMSO, being reported the rare successful for all cell types even with its potential toxicity and it being difficult to remove after thawing. On the other hand, DMSO was indicated more effective than polyvinylpyrrolidone (PVP) [113] and is now manufactured at GMP and clinical grade. Likewise, cryoprotectant media containing DMSO are available on the market such as CryoStor (BioLife Solutions). Kw et al. reported an even more high cell viability and normal cell phenotype and proliferation rate of ADSCs cryopreserved in 5% DMSO without FBS addition [114]. When adding glycerol and PL instead of FBS, high cell viability of xeno-free extracted and cryopreserved ADSCs was also demonstrated [96]. Nevertheless, despite using DMSO at clinical grade, the DMSO-containing cryomedia are differently prepared within the laboratories, non-standardized, adding to the variability of the process.

### Storage duration

ADSC use focuses on their preservation and storage possibilities; nevertheless, the optimal temperature and length of cryopreservation are particularly relevant for their cell outcomes in terms of viability and differentiation capacity [52, 104, 115–117]. For a short time (1 month), the first results did not report differences in cell viability after successive cryopreservation in − 20 °C, − 80 °C, and liquid nitrogen [117, 118]. Wolter et al. suggested better conditions in − 80 °C [115] and others favored liquid nitrogen [116]. A more recent finding indicated that ADSC viability and differentiation capabilities after 1 or 2 months cryopreservation in − 80 °C and liquid nitrogen were comparable to fresh samples [52]. Nevertheless, few investigations are conducted for the long-term cryopreservation impact on the viability and efficiency of ADSCs. Badowski et al. have reported that ADSCs and AT lose viability and differentiation capacity after storing as long as 44 months [119].

### Cell outcomes

Viability is another significant parameter reflecting cryopreservation success and it appeared closely related to the freezing rate and in some extent to thawing protocols. Indeed, post-thaw viability assessment was shown to be critical in clinical applications. Cryopreservation studies with early passages of ADSCs did not impact the immediate post-thawing viability when frozen samples were thawed at 10 °C/min in controlled rate freezer or in water bath at 37 °C [120]. Accordingly, cryopreservation has been reported to impact ADSC functionality for longer expanded ADSCs (more than 2 passages) [4].

There are evidences that the freeze-thawing process induced cell stress manifested by necrotic activity at 4–8 h post-thaw and apoptotic activity after 8–12 h thawing, leading to a time-dependent decline in viability and function at culture temperatures [121]. The need to perform viability control at different time points of post-thaw cultured cells became imminent for a more accurate assessment of cell quality and quantity. For a routine viability measurement, the trypan blue dye exclusion assay is the most commonly used. But this method is not reproducible and remains observer dependent in addition to the disadvantages of small number of cells analyzed. Many reports and consensus have suggested the use of fluorescent dyes such as 7-AAD and SYTO16 in flow cytometry analyze, as the most accurate and reliable indicators of cell viability [121, 122].

Also, to minimize osmotic shock, controlling thawing and transportation need to be more accurate. Some automated systems for vials and bags, such as ThawSTAR (BioCision) or VIA freeze (Asymptote), might help in standardizing the thawing process. These new solutions can be supported by the use of specific smart shippers (EVO Shipper, BioLife Solutions) in tracking positioning products during delivery.

## ADSC banking: adopted and emerging issues

Umbilical cord blood (UCB) banking has previously benefited from international standards implemented by both the AABB and the FACT together with NetCord (NetCord-FACT) for quality management systems and technical requirements. These standards are incorporated within their general cellular therapy standards, while FACT-NetCord CB requirements are separated but aligned with their cellular therapy standards. Such general guidelines and safeguards have been adopted despite the existence of heterogeneity and differences in ethical and legal process controlling the permissibility of ADSC use in therapeutics. As for the hematopoietic stem cells and CB, storing ADSCs for long time is currently improved using cryovials or cryobags applied for manual procedures and semi-closed or closed automated systems. However, and according to GMP requirements, stem cell banks mostly used specific cryobags to allow culture and quality control procedures such as those used for freezing and banking of CB stem cells with GMP [123]. Specific containers required for storage and shipment to the point of care are mostly identical to those used in worldwide UCB banking.

Moreover, long-term banking of BM- and UC-derived stem cells has become commonplace. A recent result reported that AT and ADSCs can be cryopreserved for up to 44 months until use [119]. However, the perspectives regarding cell viability, functionality, and integrity remain insufficient and further investigations are needed to act on the feasibility of the long-term banking. Likewise, in case of bacterial/viral infections, low temperatures would not stop proliferating infectious agents, suggesting that with consistent agents such as the coronavirus, sustainable quality control at different time point is becoming a real issue during cryopreservation.

## Product release and ADSC quality control

Most attentions should now be focused on the in-process controls and final release criteria including control production tests (cell dose, viability, immune phenotype, microbial, endotoxin, and mycoplasma testing) and functional assays (clonogenicity, trilineage differentiation, immunomodulation, and hematopoiesis regulation). This continuous testing process constituted an adequate tool to identify any dysfunctions leading to ADSC or derivative exclusion and prevent cell and time loss. Existing methods or tests for BM-MSCs and UCB are applied according to the International Pharmacopeia and could benefit to safety evaluation of biological preparations in terms of rapidity and sensitivity such as in microbiological and endotoxin testing [124, 125]. Likewise, potency and functionality assays were largely reported in the literature despite the absence of specific markers, but standardization is still lacking. Finally, concomitant environment controls might prevent any contamination risks and allowed confirmation of the graft sterility before release for transplantation.

During manufacturing, ADSCs undergo different processing conditions before being administered. As indicated in Table 1, the type of devices used to separate SVF impacted directly ADSC profile. Positive cell amounts as well as cell intensity CI were both reported different.

ADSCs could be freshly expanded, cryopreserved/thawed, and expanded or not. The expression of the classical mesenchymal markers CD44, CD73, CD90, and CD105 appeared to be irrespective of these manufacturing time points. Conversely, cryopreservation and 4 days ADSC expansion results on the increase of CD271- and CD63-positive cells. This non-classical MSC markers increase might correlate with increasing cell recovery during culture and their mRNA expressions differ in proliferative and post-proliferative ADSCs [126]. Additionally, some reports have indicated that ADSCs are negative for the monocyte/macrophage marker CD163 and their expression of the immune regulatory markers CD274 and CD276 could be predictive for their immunomodulatory potential [126–128]. Thus, culture conditions together with cryopreservation and the patient- or donor-dependent factors would impact their therapeutic outcomes and specifically their paracrine activity. ADSCs secrete largely the pro-inflammatory IL-6 factor, and this secretion could be amplified during culture expansion [4]. Combination of flow cytometry and q-PCR techniques might be more useful in characterizing clinical-grade ADSCs and complete the first criteria previously implemented by International Society for Cell Therapy (ISCT) and International Federation for Adipose Therapeutics (IFATS) as release standard. Q-RT-PCR could also be useful for testing the apoptotic activity of thawed cells.

This characterization is of interest for tracking cell behavior during manufacturing as a biomarker for cell functionality and represents a significant concern in regulating ADSCs and derivatives. Releasing clinical-grade ADSC should satisfy these parameters uniformly between GMP facilities Moreover, the recently reported side effects related to allogenic use of ADSCs have amplified the doubt on their relative safety for patients receiving manipulated ADSCs. Another concern not to be dismissed is that permitting transportation of ADSCs across borders might add to the complexity of the release testing, requiring thus the compliance with the regulations of the donor country and the receiving country. We have tried in Table 2 to track the route of ADSCs undergoing the three processing conditions before delivery. This might be useful in establishing criteria forward standardization to limit the biological and manufacturing variability.

**Table 2:** Tracking ADSCs testing from processing to delivery to the point of care

Processing conditions before administration	Location	Performed actions before delivery	Proposed actions before delivery	Actions on the point of care
Separated ADSCs (*autologous use*)	Patient bedside Cell therapy units under GLP and GCP	Viability, cell dose Functional assay (CFU-F) CD73, CD90, CD105, CD34, CD45, CD14, HLA-DR Microbial, endotoxine, mycoplasma testing	CD271, CD274, CD276, CD163, CD63 expression Cytometry and q-RT-PCR	Viability Sterility apoptotic activity
Cryopreserved and thawed (*autologous and allogenic use*)	Stem cell facility under GMP	Viability, cell dose Functional assay (CFU-F) CD73, CD90, CD105, CD34, CD45, CD14, HLA-DR Microbial, endotoxine, mycoplasma testing	CD271, CD274, CD276, CD163, CD63 expression Apoptotic activity IL-6, TNF-α and IFN expression level (q-RT-PCR)	Viability Sterility Apoptotic activity
Cryopreserved, thawed and expanded (*autologous and allogenic use*)	Stem cell facility under GMP	Viability, cell dose Functional assay (CFU-F) CD73, CD90, CD105, CD34, CD45, CD14, HLA-DR Microbial, endotoxine, mycoplasma testing	CD271, 274, CD276, CD163, CD63 expression Apoptotic activity Karyotype Migration’s genes IL-6, TNF-α, and IFN expression level (q-RT-PCR)	Viability Sterility Apoptotic activity

ADSCs can be used after separation, cryopreservation, or cryopreservation and expansion processes. During these procedures, and regarding the medical devices used to separate SVF and ADSCs and to the stem cell facilities, variabilities in cell efficiency and clinical outcomes are observed. Continuous and standardized guidelines are proposed and reinforced through a flow chart during cell processing before delivery and in the point of care

*GLP* good laboratory practices, *GCP* good cell practices, *GMP* good manufacturing practices

## Limitations of ADSC-based therapy

Spontaneous differentiation of stem cells into target cells and their migration and homing mechanisms are certainly and undeniably the tool keys in the achievement of a successful cell-based therapy. The potential benefit-to-risk in the development of ADSC therapies must be weighed and balanced at all research stages and especially during clinical translation. Functional characterization assays are evolving for a widespread clinical exploitation without documenting the cell type used regarding culture expansion and freezing (SVF, expanded or non-expanded ADSCs, number of expanding passage, frozen/thawed ADSCs). However, some limitations are still surrounding early-phase studies using ADSCs. Safety, purity, and application dose are the main concerns for an efficient therapeutic application at a large scale. In that fact, many reports have agreed for the presence of contaminating cell populations that may affect the targeted biological effects and induce potential side effects. The currently applied dose is about 1–5 × 10^6^ MSCs/kg of body weight [129–131], but their beneficial effect might be enhanced regarding time and administration route and schedule. The heterogeneity of ADSC profile and especially the use of SVF might also impact these effects, as well as the overall ADSC physiological changes observed during expansion culture. On the other side, the absence of a specific marker did not help in characterizing and standardizing ADSC clinical investigations. However, the combination of positive and negative markers should facilitate identification and selection of a closed stromal population of CD45-CD235a-CD31-CD34+ cells within SVF excluding hematopoietic and endothelial cells [86, 132, 133]. This selection should be optimized with other markers such as CD13, CD73, CD63, CD271, CD274, and CD276 for more reproducible identification or selection purposes [126, 128, 134].

Otherwise, in situ use of manipulated ADSCs is sometimes hampered by the difficulty in maintaining cell contact with the damaged tissue. Associations have emerged within latest years and used ADSCs in combination with bio-engineered materials. The most representative combination consisted of using biomaterials, growth factors, plastic support, nanostructures, polymers, etc., as a support of a tissue or organ repair based on tissue engineering [135–137]. These supports were performed to facilitate seeding of the cells and added to the difficulty in standardizing technical protocols.

In addition to the limitations already discussed [12], immunogenicity is another point of view playing a crucial role in clinical use of ADSCs. Long-term expanded ADSCs demonstrated different immunomodulatory profile. ADSC secretome is also affected by their surrounding microenvironment evidenced by differences in exosomes’ composition. The latter is mainly composed of inflammatory biomolecules, suggesting that screening of potential inflammatory factors in such cells might be a pre-requisite for their use in clinical purposes.

Genetic stability after manipulation and expansion is another major issue of the advanced application of ADSCs in therapeutics. There is controversy on the spontaneous transformation of these cells potentially by the formation of mesenchymal tissues at ectopic sites [138, 139] or accumulation of genetic alterations and malignant transformations [140–142]; these transformations seemed unrelated to the origin of MSCs. Moreover, the extremely rare malignant events reported derived from contaminating tumor cell lines [143, 144].

Consequently, the possible undesirable differentiation of ADSCs and their interaction with tumor cells raises great interest, even if the reported cases are very limited. A quantitative approach should be intended to document the functionality of ADSCs; lineage-specific gene or protein biomarkers could be used. In addition, genetic assays should be routinely integrated through conventional/molecular karyotyping before release to the point of care.

To be administered in the point of care, ADSCs or derivatives should be transferred from the facility. Actually, no specific transportation systems are available which might impact stem cell viability and proliferation. Even if liquid nitrogen is usually used for transportation, the effect on frozen cell viability cannot be neglected. Also, DMSO is not washed in the released product, reducing thus the associated therapeutic outcomes. Chu DT et al. have reported that BM-MSCs undergo similar inconvenience; however, their non-frozen transportation is completely avoided [145]. Cell-free therapy is holding great promise through using ADSC-derived exosomes. These exosomes offer crucial benefit through (i) a consistent and standardized composition using genome editing technology, (ii) increasing their release, and (iii) new possible formulations such as lyophilizates to overcome transportation and conservation disadvantages [146].

The risks associated with the medical practice and competence are the new issues that raise serious interest across cell therapy agencies. The specialty accreditation and the participation of the national government and professional authorities in settling and standardizing policies worldwide is another issue largely studied and reported by the working group of the USA [147]. Hence, the need to overcome and control secondary side effects is actually challenging. A flow chart has been developed to identify control points at any process level which appeared different from the guidelines of finished drugs. Nevertheless, the risk tier associated to this cell therapy has been limited only to the cell products and not to the route of delivery and the practitioner as it is easily applied for the investigated new drug [147]. These contradictions might raise questions on the need to refine the stem cell therapy guidance with respect to drug manufacturing.

## Refining new cell-based therapies in the context of COVID-19

### Evolving new approaches

The SARS-CoV-2 (severe acute respiratory syndrome coronavirus 2) breakdown is now the main interest of the whole world health systems. This infection is responsible of the coronavirus disease-19 so called COVID-19 and associated with a severe acute respiratory illness and multiple organ dysfunction leading to a significant mortality and a worldwide epidemic emergency. The pathogenesis of the virus is due to the presence of its angiotensin I-converting enzyme 2 receptor (ACE2), highly expressed in the lung alveolar type II cells and capillary endothelial cells [148, 149]. Other tissues also present ACE2 such as the heart, kidney, liver, and digestive organs. Additionally, alveolar cells also express the cellular transmembrane protease serine 2 (TMPRSS2) which enable the virus entering the cell membrane through priming of its spike protein [148, 150]. The viral infection results in an overreaction of the immune system as a cytokine storm. Overexpression and release of proinflammatory cytokines such as IL-1β, IL-2, IL-6, interferon α (IFNα), IFNβ, IFNγ, and monocyte chemoattractant protein (MCP)-1 lead to edema, air exchange dysfunction, acute respiratory distress, secondary infection, and multiple organ failure and even death [148, 151].

As no specific drugs or vaccines are available yet, many therapeutic plans have been proposed and most of them are supportive care rather than curative. An overview of recently investigated strategies including tackling cytokine storm, antiviral therapy, plasma from recovered patients, traditional Chinese medicine, blocking agents binding to ACE2 receptor, and vaccination have been reported [21, 152–154].

However, interesting and encouraging approaches have been realized through MSCs therapy intravenously or intrathecally administered to COVID-19 patients [17, 21, 23, 25, 27, 28, 148, 155–157]. Effectiveness and efficacy of MSCs in disease-associated inflammation and in immune diseases were well documented [158]. Global analysis of reduced mortality, patient safety, and absence or resolved side effects were demonstrated [21, 29]. The immunomodulatory effects of MSCs might be useful in attenuating or preventing the cytokine storm and outpace the evidence in treating infected patients by targeting immune cells including macrophages, dendritic cells, T and B cells, and natural killer. Adding to their action on immune cells, MSCs appeared to have an anti-microbial potential and acted through secretion of antimicrobial peptides and proteins (AMPs) and expression of indoleamine 2,3-dioxygenase (IDO) and IL-17, suggesting that these cells can increase the innate immune response to bacterial infection [159].

On October 12, more than 134 clinical trials are registered and 62 of them are using MSCs from different sources. Nineteen clinical trials with UC-MSCs are mostly ongoing where those performed with BM-MSCs and ADSCs (9 and 10 respectively) are not yet recruiting (http://clinicaltrials.gov). Autologous and allogenic MSC therapy has emerged as inhibiting of the immune system and thus treating COVID-19 pneumonia [13, 14, 21]. Assessing safety and efficacy of UC-MSCs in treating the pneumonia associated to the viral infection represents the major objective of these clinical trials [160–163]. UC-MSCs are also used in treating pneumonia in patients infected with coronavirus [164, 165].

First published results with infused umbilical cord MSCs (UC-MSCs) to critically ill COVID-19 patients present an improved therapeutic outcome [156, 162, 163]. Other strategies consisted of using ACE2^−^ UC-MSCs leading to an improved pulmonary functional activity and absence of SARS-CoV-2 nucleic acid with more benefit to elderly patients [148]. Infusion of UC-MSCs leaded to a robust anti-inflammatory activity represented by an increased number of circulating lymphocytes and a decrease in overactivated cytokine-secreting immune cells and in TNF-α in contrary to an enhanced IL-10 secretion. MSCs were largely known to auto-induce and address their microenvironment to ensure cell proliferation and tissue regeneration. It seems that UC-MSCs act similarly through paracrine effect to counteract the cytokine storm and severe inflammation, likely by protecting or rejuvenating alveolar epithelial cells [155]. Interestingly, irradiated UC-MSCs are also expected to alleviate the symptoms associated to COVID-19 pneumonia thanks to the modulating annexin-1 released by their exosomes [166]. No comparisons between MSCs sources and clinical benefit were reported in COVID-19.

Having the advantages of being in higher quantities and easy to access, ADSCs might be further presented as a promising tool in combatting COVID-19-induced pneumonia and be a part of future treatment option. ADSCs have proven efficiencies in treating pulmonary diseases in animal models through a paracrine pathway promoting thus proliferation of epithelial cells and inhibiting apoptosis [167, 168]. These cells were also reported to differentiate into type 2 alveolar cells [168]. One clinical trial is particularly on ADSC exosomes to explore the safety and efficiency of aerosol inhalations in the treatment of COVID-19-associated pneumonia. Thirteen severe cases of COVID-19 patients under invasive mechanical ventilation have received doses of allogenic ADSCs and presented improved clinical and biological outcomes [29]. Used at autologous and allogenic state, these cells have gained interest [13, 14, 19, 23, 27, 169] provided that MSCs and derivatives are performed in GMP conditions and regulated by the FDA or EMA.

### New cell- and tissue-based therapy limitations

It is conceivable that ADSCs and derivatives are a great support in treating COVID-19 patients by differentiating into adipogenic and epithelial lineages, participating in immunomodulation, angiogenesis, and anti-inflammatory responses, and improving cell regeneration. Their benefit risk still raises debate. The first criticized aspect is lung trapping as for other MSCs. However, this inconvenience is a prior advantage in treating COVID-19 patients where lung cells are infected and providing thus local immunomodulation, anti-inflammation, neo-angiogenesis, and bacterial clearance [28].

The coronavirus did not infect MSC-infused cells compared to their progeny [170]; however, many criticisms should be considered to fully attain the cell therapy efficiency and patient safety. Little is known on the behavior of the patient immunity after the coronavirus infection, on the possible recurrence regarding some factor-associated patients. ACE2 receptor is widely distributed on the kidney, liver, cardiovascular and gastrointestinal organs, white and brown AT, and cultured adipocytes [171–173]. Likewise, this receptor is the key of viral tropism in AT even if no evidences of direct infection of this tissue with the SARS-CoV-2 are reported [174]. However, transplanted patients with allogeneic BM and renal transplants were reported positive for COVID-19 and died later probably due to the extremely lower amount of T cells [175]. Prior reflections and questions rise in the case of cell therapy, but at the actual knowledge, most of them remain unresolved.

i) Presence of ACE2 and TMPRSS2 or any of their variant in tissues or in cell transplant might be a potential problem; screening of their expression levels should be a pre-requisite for both the donor and receiver for the full success of cell therapy. The immune cells, bone marrow, thymus, and lymph nodes are negative for ACE2 [170];
ii) Inactivated virus or any virus fragment might live in tissues or body fluids after healing; a control quality should take part at every point of cell processing;
iii) After primary infection, the virus might lie dormant in a specific tissue and reactivate in case of frailty or decrease in immunity like the varicella zoster virus;
iv) Use of reagents containing animal components might interact with ACE2, leading to an increase of the receptor affinity to the protein S of the dormant virus;
v) Even if stem cell products are performed in compliance with the approved guidelines from the FDA or other specific agencies, appropriate screening relative to viability, sterility, immunological profile, paracrine activity, and virus RNA testing becomes crucial for their therapeutic use.

In the proposed treating approaches, BM-MSCs are present in very low frequencies and could not be able to repair the whole damage and perform healing of the different organs’ failure. Among different stem cells, UC-MSCs seem to be preferentially used in cell-based therapies conducted for infected coronavirus patients probably for immediate access [157]. Nevertheless, ADSCs are another alternative to set up new therapeutic protocols against the inflammation storm induced by the SARS-CoV-2 favoring cell repair and regeneration. On one side, the guidelines and frameworks identified for cell- and tissue-based therapies must be reformulated to answer the new fears induced by the COVID-19. On the other side, different works reported today should lead to largely repeated investigations and well-controlled trials to confirm the beneficial effect of ADSCs. These issues are challenging and control the future cell- and tissue-based therapies.

## Discussion

Many clinical studies have been designed and conducted for a wide range of pathologies at autologous and allogenic settings. However, the variability of their therapeutic outcomes and lack of reproducibility are resulting from the absence of harmonization of their processing and their functional assessment balancing between patient safety and justification of the cell therapy. Some parameters play a critical role in achieving ADSC therapy and raises interest in terms of practicability and the eligibility of the products regarding the regulatory framework.

Anatomical site of AT collection is the first parameter to underlie regarding the number of suitable viable cells. Jurgens et al. demonstrated higher yields of ADSCs isolated from SVF in the abdominal subcutaneous than in the hip/thigh subcutaneous tissue [48] while these cells were negatively correlated to body mass index and independent of patient’s age [176]. Nevertheless, there was increasing evidence that fat source (subcutaneous or visceral) influences the proliferation and differentiation ability of ADSCs and transplantation outcome [79, 177]. Baglioni et al. have reported a significantly higher growth rate and adipogenic potential in the abdominal subcutaneous tissue [178].

Once collected, ADSC separation still raises debates. According to the FDA guidance for human cell tissue products (HCT/P), separation of non-adipocyte cell components from fat is considered as more than “minimal manipulation.” Exception could be made if only rinsing, cleansing, and sizing processing were considered, suggesting a contradictory on the SVF processing position within this regulatory. According to the EMA, ADSCs should not be cultured and isolated mechanically and used only in the subcutaneous tissue [179].

European legislation has also decided on this new advanced therapy and classified ADSCs as ATMP. Clinical use of these cells was associated to their level of substantial manipulation as a potential indicator for their functional properties [180, 181]. Uncultured or expanded ADSCs were not expected similar and might be dissociated in terms of phenotype and functional characteristics which have been largely demonstrated. Moreover, cell surface proteins involved in cell activity and the risk tier related to pathogen transmission raise additional considerations on the necessity of implementation of functional and viral testing during cell processing and especially for releasing to the point of care.

In contrary to basic cell processing, safety and quality testing are considered. Additional reagents might be used in the absence of clinical-grade ones but should be justified and controlled preventing thus transmission of animal infectious agents. Additionally, the animal host of the COVID-19 infection still remains unknown; screening of medium containing animal components such as animal serum, antibodies, or any recombinant protein should be a pre-requisite and certified before being on the market. More advancements are required focusing on the development of viable techniques to remove these animal components without the loss of valuable biological activity in the final product. Mostly, for FBS, current regulations and guidelines of biopharmaceutical products such as the European Medicine Agency EMEA (1793/02 of the proprietary medical products), the Pharmacopeia (Ph. 5 W Current edition monograph of Bovine Serum (2262), European Regulations 2005/507 for Advanced Therapies (AT), and US Code for Federal Regulation (9CFR) have recommended using of pharma-grade FBS with gamma-irradiated serum complemented with a viral test panel [45]. FBS can be replaced by PL or human serum; however, the amount of autologous serum one patient can provide is limited for a large-scale clinical expansion of ADSCs.

Another point of view to highly consider is relative to UCB collection. When pregnant women are suffering from COVID-19, it is tempting to postulate that the collected cells might not be free from viral contaminating agents and thus are not eligible for transplantation, suggesting the reinforcement of the control quality of the graft.

Another fact reinforcing the potential use of these cells is that of ADSCs can survive different freezing protocols without losing viability opening thus the way for a future large-scale cryopreservation for different therapeutic use [120, 182]. Nevertheless, viability and functionality appeared influenced by the long-term cryopreservation. Additionally, ADSC frequency should be improved without being largely and long-term expanded preventing thus any functional change or damage [4]. Elimination of CPA during freezing and thawing could be helpful to prevent any variability in cryopreserving medium preparation and practical for widely ADSC successful clinical use.

From the point of view of quality assurance programs meeting the requirement of cGMP, a uniform cell processing protocol is of critical importance. Without standardizing, all the variables may have significant protocol differences that make cross comparisons difficult. Indeed, medical devices used for SVF separation influence directly the ADSC yield and their differentiation ability. Little reports are found on the ADSC expansion at clinical grade and a specific concern should be given on harmonizing the process and to find an international consensus for a standardizing model. The feasibility of cGMP-compliant and clinical-grade ADSC preparation and banking for clinical cell transplantations should pave the way to the harmonization of the different aspects of processing and manufacturing. Perhaps, ADSCs and derivatives in accordance with GMP standards should involve several issues similar to drug manufacturing guidelines.

In the same way, we have proposed a new flow chart to perform high numbers of ADSCs in high safety and quality standard and preventing at least the assessment variability. In the case of using uniformed medical devices within GMP facilities, it is tempting to speculate that the reproducibility of ADSC efficacy will result from these standard operating procedures. Hence, achieving their adequate clinical effect will remain patient’s associated factors providing the presence of specialized practitioner. Specialty societies have followed and considered all these questions for BM and UCB transplantations leading to accreditations of stem cell banks which benefit to the manufacturing practices in different facilities operating in the field. Bringing ADSCs and derivatives into the market should take the same way.

However, and with regard to patient security, a special insight should be performed on the presence of COVID-19’s mRNA or derived proteins (protein S) even inactivated within the donor tissues. There are evidences arguing a viral tropism of AT and the presence of ACE2 in adipocytes [16]. Despite the promising alternative offered by using ADSCs in COVID-19, the relationship between adipocyte hypertrophy mediated by ACE2 receptors and COVID-19 might imbalance their benefit as a potential widespread therapeutic tool. Thus, viral compounds might reside or resist within AT leading to the necessity to set up viral identification from fat collection and might be during the whole processing of all stem cell-based products. Emerging strategies should take place to investigate the extent of this virus and the different ongoing vaccine testing on human tissues and on the operating procedures. Cell and tissue transplantation landscape is upset and is facing a new challenge with the SARS-CoV-2 virus breakdown.

## Conclusion

ADSCs have proven efficiency in regenerating damaged tissues in vitro and in vivo. However, their self-renewal and multipotency behavior remained the focus of the success of their therapeutic use as it presents multiple variabilities preventing comparisons and practicability. Scientific, practitioner, and specialty societies should converge efforts on the continuously optimized parameters such as sourcing, cell dose, cryopreservation, banking, and transplantation methods altogether with functional assessments. Optimizing and standardizing new guidelines relative to this process are really challenging as the scope of the COVID-19 remains unknown.

## Data Availability

Not applicable.

## Abbreviations

ADSCs: Adipose-derived stem cells
AT: Adipose tissue
HCT/P: Human cells, tissues, or cellular or tissue-based products
AMTPs: Advanced therapy medicinal products
BM: Bone marrow
UCB: Umbilical cord blood
GMP: Good manufacturing practices
FDA: Food and Drug Agency
CPA: Cryoprotective agent
DMSO: Dimethyl sulfoxide
FBS: Fetal bovine serum
HS: Human serum
MSCs: Mesenchymal stem cells
PBS: Phosphate-buffered saline
hPL: Human platelet lysate
SVF: Stromal vascular fraction
COVID-19: Coronavirus disease-19
